# Gelatin–St. John’s Wort Oil Matrices: Material Properties for Potential Biomedical Applications

**DOI:** 10.3390/polym18111360

**Published:** 2026-05-30

**Authors:** Mehlika Karamanlioglu

**Affiliations:** Biomedical Device Technology Department, Istanbul Gelisim University, 34310 Istanbul, Türkiye; mkaramanlioglu@gelisim.edu.tr

**Keywords:** biomaterial, biomedical application, gelatin, mechanical properties, physicochemical properties, St. John’s wort oil, wound dressing

## Abstract

This study investigates physicochemical, mechanical, and thermal effects of St. John’s wort (JW) oil on gelatin-based films for potential biomedical applications as there is limited research on gelatin biomaterials containing JW oil as sole bioactive component. Transparent films were fabricated at gelatin:JW oil ratios of 20:0, 20:1, 20:5 (*w*/*w*) designated as JW-0, JW-1, JW-2, respectively, via solution casting. Gas chromatography revealed that JW oil is rich in unsaturated fatty acids, predominantly linoleic and oleic acids, while FTIR confirmed their successful integration into the gelatin matrix through the fatty acid peak at 1743 cm^−1^. Oil droplets, increasing with oil content was shown by SEM. JW oil improved water durability by reducing water aging by up to 8%. JW oil acted as a plasticizer, raising elongation at break (EAB) from 188% in JW-0 to 231% and 209% in JW-1 and JW-2, respectively. DSC indicated a higher T_max_ in JW-1 (116 °C) compared to JW-2 (110 °C), evidencing better thermal stability. In conclusion, JW oil can be effectively incorporated into gelatin as a single active component. Specifically, JW-1 formulation achieved an optimal balance between mechanical and structural integrity, flexibility, and thermal stability, underscoring its potential as a cost-effective, bioactive wound dressing material.

## 1. Introduction

Traditional wound dressing materials primarily serve as protective barriers but often lack mechanical and bioactive properties [1]. In recent years, polymer-based advanced wound dressings that incorporate bioactive compounds and biodegradable matrices have garnered significant attention due to their potential to accelerate tissue regeneration, mitigate infection risks, and enhance patient comfort [1,2]. Among these materials, gelatin-based films have emerged as promising candidates due to gelatin’s excellent film-forming properties, biocompatibility, bioadhesiveness, hemostatic nature, flexibility, moisture-retention capability, and cost-effectiveness [3,4,5,6,7,8].

In the literature, gelatin films are often combined with other polymers to incorporate different types of herbal oil for developing wound dressing materials [9,10,11,12,13]. Therefore, in our previous studies, we incorporated coconut oil to pure gelatin films and showed that coconut oil improved thermal stability, wound healing and some mechanical properties of the gelatin films [14,15]. Since the oil content enhanced properties of gelatin films, in the present study, we assessed the influence of another herbal oil, St. John’s wort (JW) oil, on material properties of gelatin films. Moreover, since gelatin is obtained through the hydrolysis of collagen, and JW oil has been reported to increase collagen levels [16], their combination may have implications for wound healing when JW oil is incorporated into gelatin-based biomaterials.

JW oil, extracted from *Hypericum perforatum* plant, has bioactive hypericin and hyperforin components which have analgesic, anti-inflammatory, and wound healing effects [16,17,18,19,20,21,22,23,24,25]. *H. perforatum* extract was shown to have antimicrobial activity against some bacteria [26,27] and in vitro anti-cancer activity [27,28]. Therefore, JW oil, which has been widely used in the food industry [7,8], has been used in medicine and cosmetics as well [16,17,25] and it is suggested as a remedy for various dermatological conditions including wound healing [29].

Topical administration of JW oil is determined to be effective in wound healing of second-degree burns [24]. Similarly, topical JW oil application on burn wounds improved epithelialization [30]. In some types of ulcers, JW oil has been shown to heal wounds upon application [21]. In another study, JW oil was shown to heal wounds in vivo when used during the early stages [31]. An ointment having natural ingredients including JW oil was also shown to enhance wound healing [32].

JW oil was also incorporated in other polymers for biomedical applications as well. Wound healing effect of JW oil was shown when used in chitosan films [33], chitosan-based hydrogels [19], cryogels [34], and chitosan nanoparticle/agarose films [35]. Physical and antibacterial effects of JW oil on alginate [36], polyvinyl alcohol (PVA)/chitosan [37], and gelatin-chitosan [38]-based wound dressing materials were also demonstrated. Another study focused on developing gelatin nanofibers cross-linked by tannic acid, loaded with JW oil extract and vitamin A palmitate for wound dressing purposes [39]. Thermoplastic polyurethane-gelatin mats with JW oil cross-linked by tannic acid were shown to be antibacterial materials with wound healing activities and their structural modifications were analyzed [40]. Biocompatibility and antibacterial effect of JW oil were shown in vitro when used in bio-based sponges [41] and also when used in synthetic PCL nanofibers [42]. When composite nanofiber membranes of polyethylene oxide (PEO) and polyvinyl alcohol (PVA) were prepared with several plant extracts from different plants including *H. perforatum*, wound healing properties of the membranes were demonstrated with improved mechanical properties [43]. JW oil with another plant extract incorporated in thermoplastic polyurethane/polylactic acid blend foams displayed in vitro wound healing and non-cytotoxicity [44]. JW oil addition to methyl cellulose/okra mucilage composite films containing gentamicin decreased tensile strength but increased wound closure rates in vitro [45].

There is limited research on the integration of solely JW oil into gelatin matrices and on how JW oil mechanically and physicochemically modifies gelatin-based films. Most of the studies summarized above focused on a limited number of structural and mechanical properties of JW oil-containing biomaterials while emphasizing the potential wound healing properties of JW oil as a wound dressing component. Therefore, this study primarily focuses on emphasizing the structure–property relationships of JW oil-modified gelatin films, which is a necessary prerequisite before biological validation. Given the well-documented pharmacological properties and wound healing properties as summarized above, JW oil incorporation in gelatin matrices holds the potential to create an advanced wound dressing with both structural integrity and therapeutic benefits. In contrast to previous studies that incorporated JW oil into composite films composed of various polymers [40,43,44,45] and other plant extracts [43,44] or into films containing both gelatin and other biopolymers such as chitosan [38] or into gelatin nanofibers containing additional components such as tannic acid and vitamin A palmitate [39], this study exclusively focuses on gelatin-based films and the sole influence of JW oil on the material properties of these films. Although previous studies demonstrated promising biomedical and antibacterial properties, the simultaneous presence of multiple polymers and/or additional bioactive compounds made it difficult to identify the direct contribution of JW oil to the structural, physicochemical, thermal, and mechanical characteristics of the resulting materials. In particular, interactions arising from secondary biopolymers or active additives may mask or modify the effects of JW oil within the gelatin matrix. Therefore, this study uniquely investigates JW oil in a film composed solely of gelatin, thereby eliminating interactions with other biopolymers and enabling a clearer structure–property relationship analysis to isolate the sole effects of JW oil. Additionally, using a single polymer simplifies manufacturing and reduces costs, making large-scale production more feasible. By incorporating different amounts of JW oil, this research uniquely investigates its physicochemical, mechanical, and thermal effects on gelatin films, providing new insights into its potential as a cost-effective primary wound dressing material for acute wounds.

## 2. Materials and Methods

### 2.1. Materials and Chemicals

Bovine skin gelatin powder with an approximate Bloom number of 240 g was purchased from Lokman Hekim (Ankara, Türkiye). St. John’s wort oil was acquired from Arifoğlu (İstanbul, Türkiye). Glycerol, a plasticizing agent, was obtained at 98% reagent grade from ISOLAB (Eschau, Germany). Commercial-grade emulsifier Tween 80 (polysorbate 80) was purchased from Sigma-Aldrich (Eschau, Germany).

### 2.2. Gas Chromatography

Fatty acid composition of JW oil was analyzed using gas chromatography (GC Perkin Elmer, Autosystem GLX, Shelton, CT, USA) at TÜBİTAK Marmara Research Centre according to ISO 12966-2:2011 [46]. TotalChrom Navigator (version 6.3) was used for data collection and quantification.

### 2.3. Fabrication of Gelatin–JW Oil Films

Solution casting method [14] was employed to prepare gelatin-based film samples. Powder gelatin, 20% (*w*/*w*), was dissolved in distilled water to form film-forming solution (FFS) at 50 °C on a magnetic stirrer at 1000 rpm for 30 min. Glycerol at 8% (*w*/*w*) was then added to FFS containing dissolved gelatin at 50 °C as a plasticizer. JW oil at 20:0, 20:1, 20:5 (*w*/*w*) mass ratio (gelatin:JW oil) corresponding to JW-0, JW-1, JW-2 films, respectively, was added into FFS in separate beakers. As an emulsifier, 3% (*v*/*w*) Tween 80 was added to each FFS, including JW-0, to enable the homogeneous dispersion of JW oil within the FFS. Tween 80 was incorporated into all films regardless of the presence of JW oil. Therefore, the control film had all components except the oil itself, enabling a clearer assessment of the sole effect of JW oil incorporation on film properties. Solutions of 25 mL from JW-0, JW-1 and JW-2 were cast into 80 × 150 mm glass dishes. Films dried at room temperature at a relative humidity (RH) of 40 ± 4%. All the films were kept under the same conditions of 25 °C ± 4 and 40 ± 4% RH for at least one day prior to any analyses.

### 2.4. Scanning Electron Microscopy (SEM)

Zeiss EVO^®^ LS 10 (Carl Zeiss AG, Oberkochen, Germany) was used to evaluate the microstructure of JW-0, JW-1 and JW-2 films at an acceleration voltage of 10 kV. Prior to examination by SEM, all films were dried and gold–palladium coated. The surface and cross-section of the films were analyzed at a magnification of 100–600×.

### 2.5. Determination of Moisture Content, Water Uptake, Water Aging Behavior of the Films

Triplicate samples of JW-0, JW-1, and JW-2 films were used to determine their moisture content, water uptake, and water aging behavior. The standard deviation was used to calculate the standard error of the mean. To determine the moisture content of each film (1), the initial mass (m_i_) of each film was measured. Then, the films were dried to a constant mass at 30 °C until constant weight (m_d1_).

To calculate the water uptake of the films (2), the initial mass of each film (m_i_) was measured. Then, the films were immersed in water separately and removed periodically at 30 min time interval to measure the final mass of the soaked films (m_s_) until the films disintegrated. Excess water was blotted out from the films at each time interval.

After the water uptake test, the remaining fragments of the soaked films were recovered by filtration and dried to a constant weight at 30 °C ± 4 (m_d2_) for the determination of the water aging of the films (3) [6].

The equations below were used to calculate moisture content (1), water uptake (2) and water aging (3) of the films [6,14]:

(1) $$\mathrm{Moisture}\;\mathrm{content}\;(\%)\;=\;\frac{m_{i}-m_{d1}}{m_{i}}\times 100$$

(2) $$\mathrm{Water}\;\mathrm{uptake}\;(\%)=\frac{m_{s}-m_{i}}{m_{i}}\times 100$$

(3) $$\mathrm{Water}\;\mathrm{aging}\;(\%)=\frac{\;m_{s}-m_{d2}}{m_{s}}\times 100$$

where

m_i_: initial mass;

m_d1_: mass of the dried samples;

m_s_: mass of the samples after being soaked in water;

m_d2_: mass of the dried samples after being soaked in water.

### 2.6. Mechanical Properties

The mechanical properties of JW-0, JW-1, and JW-2 were determined in triplicate according to ASTM D882-18 using Zwick Z250 Universal Testing Machine (Zwick/Roell GmbH & Co. KG, Ulm, Germany) equipped with a 1 kN load cell and a crosshead speed of 200 mm/min at 23 ± 2 °C at TÜBİTAK Marmara Research Centre [47]. Each film’s tensile strength (TS) and elongation at break (EAB) values were obtained. The initial slope of stress–strain curves was used to calculate Young’s modulus (YM) for each film. The thickness of the films was measured with a manual digital micrometer (Mitutoyo, Kawasaki, Japan).

### 2.7. Fourier Transform Infrared Spectroscopy (FTIR)

Structural bonds and interactions of JW-0, JW-1, and JW-2 were determined using Perkin Elmer Spectrum 400 FTIR spectrophotometer (PerkinElmer, Shelton, CT, USA) with attenuated total reflectance (ATR). Transmittance mode was used to scan the spectra in the wavenumber range of 650–4000 cm^−1^ with a resolution of 2 cm^−1^.

### 2.8. Differential Scanning Calorimetry (DSC)

Perkin Elmer Pyris 1 Differential Scanning Calorimeter (PerkinElmer, Shelton, CT, USA) was used for thermal analysis of 5 to 10 mg of JW-0, JW-1 and JW-2 samples in triplicate. Heating was at 10 °C/min over the temperature range from 10 °C to 250 °C. Glass transition temperature (T_g_) and maximum melt-like transition temperature (T_max_) of films were determined.

### 2.9. Statistical Analyses

IBM SPSS Statistics 24 Version was employed for statistical analyses of mechanical properties and thickness of the samples. To determine the statistical significance of any differences in thickness, tensile strength (TS), Young’s modulus (YM), and elongation at break (EAB) among JW-0, JW-1, and JW-2 films, one-way ANOVA (Analysis of Variance) and Tukey’s HSD (honestly significant difference) tests were used. The significance threshold was set at a *p* value of less than 0.05. Each film’s result for each property is presented with a mean value and a standard error of the mean, which was calculated from the standard deviation.

## 3. Results and Discussion

### 3.1. Fatty Acid Composition of JW Oil

Fatty acid composition of extracted JW oil used in this study is presented in Table 1 with their percent concentrations. The most dominant fatty acid in JW oil used in this study was linoleic acid (56%), followed by oleic acid (27%), palmitic acid (9%), and stearic acid (3%). Both linoleic acid and oleic acid are unsaturated fatty acids, whereas palmitic and stearic acid are saturated fatty acids. Linoleic acid is an omega-6 fatty acid and oleic acid is an omega-9 fatty acid. The wound healing effect of both lineolic acid and oleic acid was shown in vivo since both acids influence the release of pro-inflammatory cytokines on cutaneous wounds [48]. Oleic acid was shown to induce wound healing faster than linoleic acid and linolenic acid [49], which was 0.15% of JW oil used in this study. Furthermore, linoleic acid and linolenic acid are precursors of arachidonic acid, which was approximately 0.3% in the JW oil. Notably, arachidonic acid is also a precursor of certain molecules that are involved in the mediation of inflammation [50].

### 3.2. Film Formation

**Figure 1:**
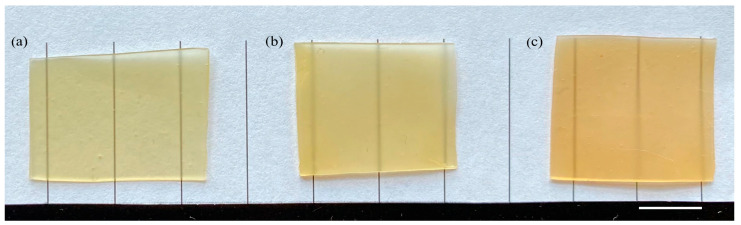
(**a**) JW-0, (**b**) JW-1, and (**c**) JW-2 films at gelatin: JW oil mass ratios of 20:0, 20:1, and 20:5 (*w*/*w*), respectively. Scale bar represents 1 cm.

JW-0, JW-1 and JW-2 films obtained by solution casting method had smooth, continuous, and homogeneous surfaces without any immiscible oil drops or pores on the surface (Figure 1). All the films were transparent which enables observation of the healing progress without removal of the dressing material [51]. However, the sheerness decreased with increasing oil content, as the redness of the JW oil became more visible at higher concentrations. The red color of JW oil comes from keeping the dried yellow flowers of the *H. perforatum* plant under sunlight for a month [24,34], and it was observed only in JW-2 films since JW-2 had more JW oil content.

### 3.3. Microstructure of Films

**Figure 2:**
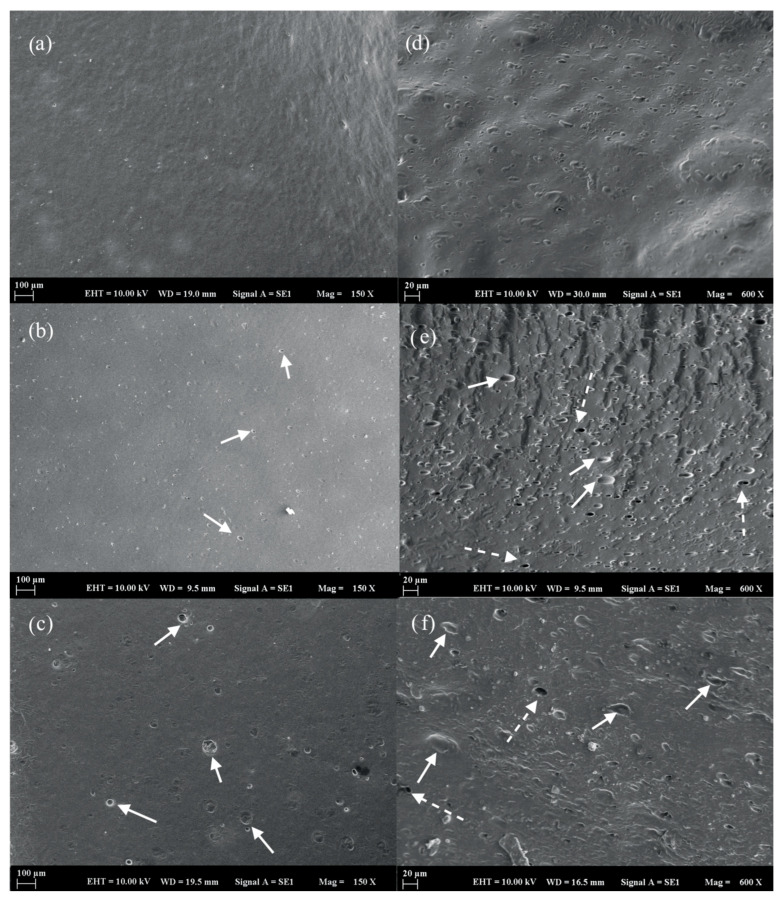
SEM images of surface of (**a**) JW-0, (**b**) JW-1 and (**c**) JW-2 films; and cross-section of (**d**) JW-0, (**e**) JW-1 and (**f**) JW-2 films. Oil droplets are indicated by arrows. Pores are indicated by dashed arrows.

The microstructure of films was analyzed by SEM as shown in Figure 2. JW-0 film had smoother and more homogeneous surface and cross-section compared to oil-containing films (Figure 2a,d).

Surface of JW-1 film was heterogeneous since it contained small oil droplets of about 10–20 µM (Figure 2b). Cross-section of JW-1 also showed droplets of about the same size (Figure 2e). Since oil droplets were more in the cross-section of JW-1, oil droplets were embedded more in the gelatin matrix rather than being on the surface. Pores about 10 µM wide were also observed in the cross-section of JW-1.

The surface and cross-section of JW-2 films (Figure 2c,f) showed bigger droplets than JW-1 films. On the surface, a larger amount of oil droplets about 30–80 µM in size were observed (Figure 2c). Embedded oil droplets of ~30–40 µM were also observed along with some pores ~20 µM in the cross-section of JW-2 (Figure 2f). Oil droplets observed on JW-2 had more irregular shape than the oil droplets on JW-1 film which had an ellipse structure.

In general, oil-containing films were more heterogeneous due to the presence of pores and hydrophobic oil droplets. As the amount of oil increased in the films, the size of the oil droplets and pores increased as well. Similarly, in the literature, regardless of the oil type, microstructure of oil-containing protein-based films was observed to have heterogeneous surfaces due to the presence of oil droplets and pores [14,15,52,53,54,55]. Although the films contained emulsifier, immiscible and hydrophobic oil droplets were separated possibly during casting [15,52,54]. Pores were also formed in other oil-containing films due to the separation of hydrophobic oil droplets during casting of the films [55,56]. Oil droplet size also induced pore formation in gelatin films as well [55].

### 3.4. Moisture Content, Water Uptake and Water Aging Behavior of the Films

Moisture content of JW-1 and JW-2 films were about 2% and 12% less than JW-0 films, respectively, due to the hydrophobicity of the oil content (Table 2). Moreover, as the oil content increased, moisture content decreased when JW-1 and JW-2 were compared.

**Figure 3:**
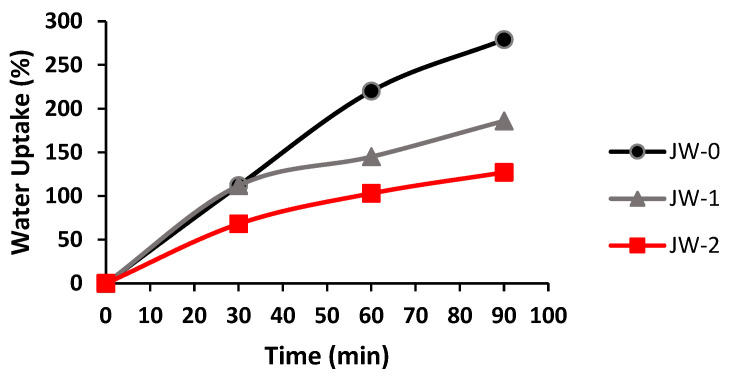
Water uptake of JW-0, JW-1 and JW-2 films.

Water uptake, which is crucial for wound dressings to absorb exudates from the wound site [57], increased over time for all films; however, the capacity of water uptake decreased with increasing oil content (Figure 3). Therefore, the highest water uptake percentage was recorded for JW-0 films, which was about 280%, while the percentages were about 186% and 127% for JW-1 and JW-2 films, respectively. Although the water uptake percentages of JW oil-containing films decreased compared to pure gelatin films, the values still remained above 100%. Therefore, JW oil incorporation did not eliminate the fluid absorption capability of the films, which is important for wound exudate management. In particular, JW-1 films are more suitable for wound exudate absorption than JW-2 due to their higher water uptake capacity (186%). Moreover, a moderate reduction in water uptake may help prevent excessive swelling and loss of structural integrity during use, which may also contribute to the lower water aging values observed in JW oil-containing films. In this regard, JW-0 films exhibited the highest water aging percentage (~89%) compared with JW-1 (~83%) and JW-2 (~80%) films (Table 2). Wound dressings require low water aging percentages [6] and JW oil improved water aging behavior of gelatin films by reducing it by up to 8% for JW-2 films. Therefore, JW incorporation improved dressing integrity during storage and use. In another study, the water uptake of pure gelatin films was reported to be about 200%; however, the films dissolved under 10 min in water [6]. Compared to that study, JW oil incorporation improved the durability of gelatin films in aqueous conditions without hindering their water uptake ability.

### 3.5. FTIR

**Figure 4:**
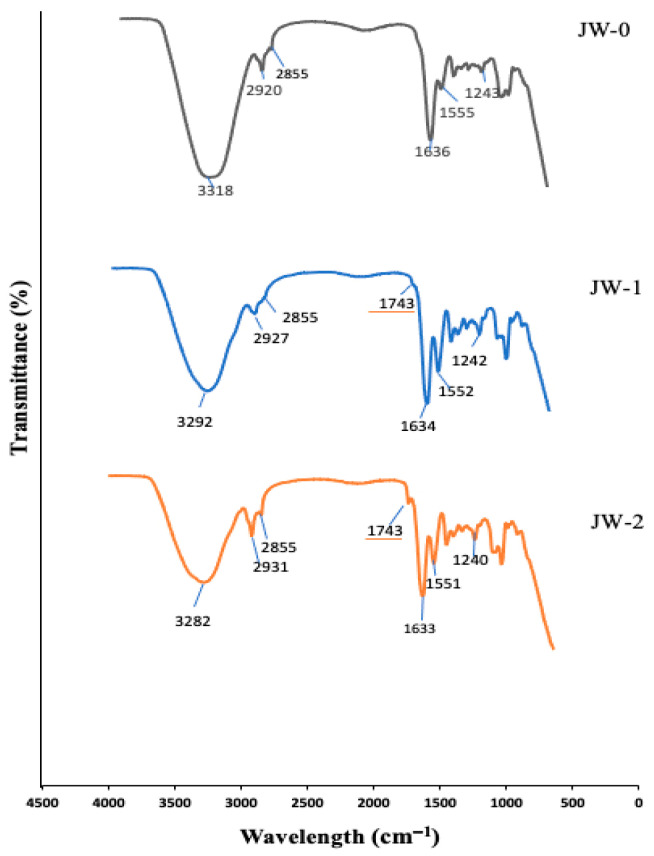
FTIR spectra of JW-0, JW-1 and JW-2 films. The JW oil-related peak is underlined.

FTIR spectra of JW-0, JW-1 and JW-2 are compared in Figure 4. The main transmittance peak of protein was positioned at 3318 cm^−1^ in JW-0 films. This peak is related to the amide-A band corresponding to N-H stretching vibration and hydrogen bonding of NH groups among protein molecules [58]. Incorporation of JW oil shifted the amide-A band to lower frequencies, i.e., 3292 and 3282 cm^−1^ in JW-1 and JW-2 films, respectively. According to the literature, shifts in the amide-A band toward lower frequencies may indicate increased hydrogen bonding involving NH groups in the peptide chains of gelatin [58].

The peak situated at 2920 cm^−1^ in JW-0; at 2927 cm^−1^ in JW-1 and at 2931 cm^−1^ JW-2 films; and the peak at 2855 cm^−1^ in all samples are related to C-H stretching [59]. The peaks observed at ~2927 cm^−1^ and ~2855 cm^−1^ were also characterized in gelatin films containing olive oil [59] and chitosan cryogels containing JW oil [34]. The same peaks were also identified in the FTIR spectrum of linoleic acid [60] and oleic acid [61] as the characteristic CH_2_ stretching vibrations [62].

The amide-I band, which is associated with hydrogen bonding between C=O groups of protein molecules, was positioned at 1636 cm^−1^ [58] in JW-0 and 1634 cm^−1^ and 1633 cm^−1^ in JW-1 and JW-2 films, respectively. According to the literature, shifts in the amide-I band toward lower frequencies may indicate increased hydrogen bonding involving C=O groups in gelatin [58]. The shifts in amide-A and amide-I bands toward lower wavenumbers may suggest changes in intermolecular interactions, including possible hydrogen bonding within the gelatin matrix. However, considering the oil-containing and heterogeneous nature of the films, these shifts may also be influenced by physical interactions with JW oil, changes in moisture content, and oil-induced plasticization effects, where increased chain mobility and loosening of the gelatin network may cause small FTIR band shifts while simultaneously decreasing TS values.

The amide-II band was observed at 1555 cm^−1^ in JW-0 and it shifted to 1552 cm^−1^ and to 1551 cm^−1^ in JW-1 and JW-2 films, respectively, with increased amplitude. As the amount of oil increased, the amplitude of the amide-II peak increased, showing the increase in free water due to bonded proteins [63]. The amide-III peak was situated at 1243 cm^−1^ in JW-0; however, it shifted to lower frequencies of 1242 and 1240 cm^−1^ in JW-1 and JW-2 films, respectively. In conclusion, the main gelatin structure was not affected by JW oil addition since the characteristic peaks of gelatin, i.e., amide-A and amide-I-III bands, were still present in JW films. These bands only shifted to lower frequencies upon incorporation of JW oil, indicating hydrogen bonding among protein molecules [58]. Similarly, in our previous studies, incorporation of coconut oil to gelatin films shifted most amide bands to lower frequencies [14,15].

Apart from the characteristic protein peaks, a new peak appeared only when samples contained JW oil, showing the successful JW oil incorporation. This transmittance peak at 1743 cm^−1^ is associated with the carbonyl group (C=O) of the carboxylic acids, i.e., fatty acids, of JW oil [34,60] which are also shown by gas chromatography. This data is consistent with SEM since oil droplets were observed in gelatin matrices via SEM. The amplitude of this peak increased as the oil amount increased. Similarly, in another study, olive oil droplets were shown on gelatin films with confocal laser scanning microscopy and the characteristic peaks of oil were shown in FTIR spectra [59]. Moreover, when JW oil was added to chitosan cryogels, the same peak appeared in only JW-containing wound dressing materials [34]. In another study, C=O stretching vibration of carboxylic acid of neat linoleic acid was observed at 1719 cm^−1^ [60].

### 3.6. Effect of JW Oil on Mechanical Properties

**Figure 5:**
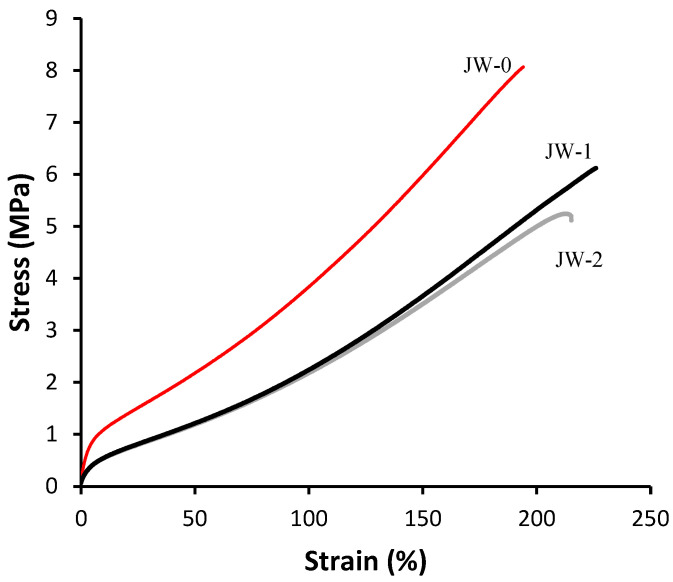
Stress–strain curves of JW-0, JW-1 and JW-2 films.

The effect of embedded JW oil in gelatin matrices was determined by measuring mechanical properties of the fabricated films as well. Thickness values of the films, which were similar (*p* > 0.05), were also measured to calculate mechanical properties. Stress–strain curves for JW-0, JW-1, JW-2 films are shown in Figure 5. The shape of the curves, which is typical for gelatin films [64], is similar in all films. These curves are characterized by an initial elastic region in which the slope was used to calculate YM and it was at a higher modulus in JW-0 films. The elastic region was then followed by yield point and plastic deformation in all films. After the maximum load, corresponding to TS, films ruptured. Similar modulus was observed in the curves of JW-1 and JW-2 films; however, EAB of JW-2 films was lower than JW-1 films as these films ruptured before JW-1 films during testing.

Also, as seen in Table 3, JW oil addition significantly decreased TS and YM and increased EAB when compared to JW-0 (*p* < 0.05). Moreover, increasing oil content decreased TS more since TS of JW-2 films was significantly lower than that of JW-1 (*p* < 0.05). YM of JW-1 and JW-2 were similar (*p* < 0.05) due to the similar modulus of the initial elastic region of curves as seen in Figure 5.

Heterogeneous microstructure of oil-incorporated films observed by SEM and FTIR would cause discontinuities in the film matrix, decreasing TS and YM of oil-incorporated films. TS of human skin ranges between 5 and 30 MPa [65], and since TS of JW-1 and JW-2 were 6.3 MPa and 5.3 MPa, respectively, they can still be suitable as wound dressing materials. Similarly, previous studies reported that oil incorporation decreased the TS and YM values of protein- and polysaccharide-based films [15,66,67]. More specifically, JW oil incorporation decreased TS and YM while increasing EAB of chitosan films [33] and methyl cellulose/okra mucilage composite films [45]. Moreover, when 1.5% (*v*/*v*) JW oil was incorporated into chitosan films, the Young’s modulus decreased to approximately 6 MPa [33]. In contrast, despite containing higher amounts of JW oil, the gelatin-based films prepared in the present study maintained a higher Young’s modulus of approximately 10 MPa, indicating comparatively better mechanical integrity.

Furthermore, JW oil significantly increased the EAB of gelatin-based films, which is necessary for wound dressing applications on irregular wound surfaces and mobile anatomical regions, particularly near joints [68]. The presence of unsaturated fatty acids in JW oil, particularly linoleic acid, may increase chain mobility due to their less rigid structure, thereby contributing to the improved flexibility and altered intermolecular interactions within the gelatin matrix. Furthermore, the increased flexibility of JW oil-containing films may, potentially reduce the risk of cracking during application.

Although JW oil incorporation reduced TS and YM values, the films still maintained tensile strength values within the lower range reported for human skin [65], suggesting that they may retain sufficient mechanical integrity for handling and wound coverage during use.

The plasticizing effect of oil content was shown to increase flexibility of the films in other studies as well [4,53,56,66,69,70,71]. When JW oil amounts are compared, the lower amount of JW oil as observed in JW-1 films caused a significantly higher EAB than JW-2 (*p* < 0.05). Similarly, in another study, when chitosan films contained 0.5% JW oil, its EAB was more than the ones that contained 1% and 1.5% JW oil [33]. Therefore, JW-1 films may adapt more easily to skin surface. These results are consistent with our previous study, in which the gelatin film with the lowest coconut oil content had more flexibility than the films with increased oil content [15]. Since TS of JW-1 was significantly more than JW-2 (*p* < 0.05), it can be concluded that films with lower JW oil content had much more improved mechanical properties than JW-2 films, which can be attributed to the less heterogeneous microstructure of JW-1 since it had smaller sizes of pores and oil droplets observed by SEM (Figure 2). Tween 80 is a non-ionic surfactant with relatively low toxicity and may also contribute to increased EAB values due to its surfactant and plasticizing effects. For this reason, Tween 80 was intentionally included in JW-0 films as well.

### 3.7. Thermal Properties

**Figure 6:**
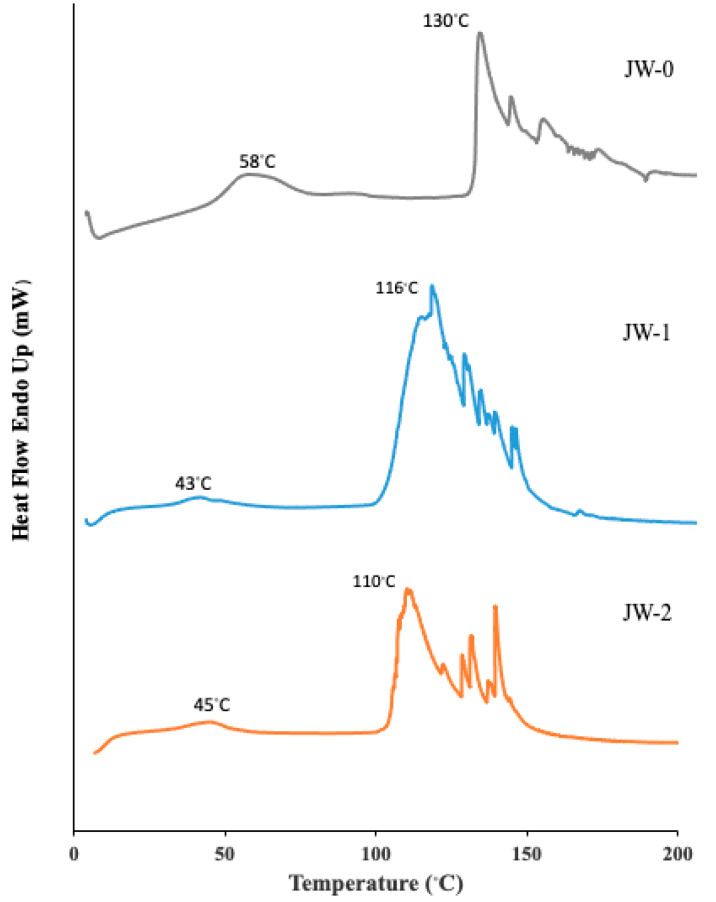
Thermograms of JW-0, JW-1 and JW-2 films.

The effect of JW oil on thermal properties of gelatin films was determined by comparing the thermograms of films (Figure 6). The first endothermic peak of JW-0 film was at about 56 °C, which corresponds to the glass transition temperature, T_g_, above which gelatin becomes viscous. This endothermic peak shifted to lower temperatures to 43 °C and 45 °C in JW-1 and JW-2 films, respectively, indicating lower chain stiffness and lower thermal stability of gelatin films [58,72] upon JW oil incorporation. Plasticizers in gelatin-based films were previously shown to shift T_g_ to lower temperatures [73]. Moreover, gelatin films with lower T_g_ had reduced mechanical strength and stiffness [58]. Therefore, JW- incorporated films had lower TS and YM due to their reduced thermal stability but higher elasticity due to lower chain stiffness [58,73,74].

Endothermic peaks observed after T_g_ are associated with helix–coil transition showing the melt-like transition of gelatin in the films [73]. Multiple endothermic peaks after T_g_ was also observed in our previous studies, when coconut oil was added to gelatin films [14,15]. Melting and recrystallization of various sizes and types of crystalline regions may have caused multiple peaks in the thermograms [15,75]. The maximum endothermic peak (T_max_), corresponding to the temperature of helix–coil transition and disruption of the ordered molecular structures [58], was observed at 130 °C in JW-0 films but it decreased to 116 °C and 110 °C in JW-1 and JW-2 films, respectively. Lower T_max_ of JW incorporated films shows lower molecular order of gelatin films. Therefore, JW oil incorporation made gelatin films more amorphous and thus weaker, causing decreased TS and YM but increased EAB. However, T_max_ of JW-1 was at a higher degree than JW-2; therefore, JW-1 films had more molecular order than JW-2 films, improving its mechanical properties.

The incorporation of JW oil affected the microstructure of gelatin films by forming dispersed lipid droplets and pores as observed in SEM images. These structural changes may have reduced increased chain mobility. This was supported by lower T_g_, T_max_, and also by YM values together with the improved flexibility of the films.

Although JW oil reduced T_g_ and T_max_ of gelatin films, our previous studies [14,15] demonstrated that coconut oil incorporation increased both T_g_ and T_max_ of gelatin films. This indicates that oil type, i.e., the fatty acid composition/saturation, significantly impacts the thermal properties of the films.

## 4. Conclusions

To emphasize the functional role of JW oil as the sole bioactive component in gelatin-based wound dressings, physicochemical, mechanical, and thermal properties of gelatin films incorporated with JW oil were characterized. Gas chromatography analysis revealed that JW oil used in this study was rich in unsaturated fatty acids, particularly linoleic acid (56%) and oleic acid (27%), both associated with wound healing properties. JW oil was successfully incorporated into gelatin-based films by the solution casting method. The hydrophobic oil content did not hinder water uptake and improved the water aging behavior of gelatin films, which are important properties for wound dressing applications. SEM analysis revealed the presence of oil droplets and pores within the gelatin matrix, and the size of these structures increased with increasing JW oil concentration. FTIR results were consistent with SEM and gas chromatography findings as the transmittance peak of fatty acids at was present in the FTIR spectra of JW incorporated films. JW oil incorporation increased the flexibility of the films due to its plasticizing effect, as evidenced by increased elongation at break values. However, increasing oil concentration also reduced tensile strength, Young’s modulus, T_g_, and T_max_ values, indicating reduced molecular order and thermal stability at higher oil contents. Nevertheless, films containing lower JW oil concentration (JW-1) exhibited more balanced mechanical and thermal properties compared to JW-2 films, suggesting that excessive oil incorporation may negatively affect film integrity and stability.

This study demonstrated that JW oil can be utilized as the sole bioactive component of gelatin films, thereby enabling their potential application as primary wound dressing materials for acute wounds. In particular, JW-1 films with a gelatin:JW oil ratio of 20:1 (*w*/*w*) exhibited much improved mechanical and thermal properties, as well as a more homogeneous microstructure, compared to JW-2 films with a higher JW oil content at a gelatin:JW oil ratio of 20:5 (*w*/*w*). Consequently, JW-1 films can be further explored through cytotoxicity, cell compatibility, and wound healing studies to validate the biological activity of JW oil in gelatin-based matrices, as JW-1 films demonstrated more advantageous physicochemical and mechanical properties for potential wound dressing applications. The present study primarily focused on establishing the structure–property relationships of JW oil-modified gelatin films as a prerequisite for subsequent biological validation studies.

## Figures and Tables

**Table 1:** Fatty acid composition of JW oil used in this study.

Fatty Acid	Percentage (%)
Linoleic Acid (C18:2n6c) *	56.42
Oleic Acid (C18:1n9c)	27.52
Palmitic Acid (C16:0)	9.44
Stearic Acid (C18:0)	3.17
Behenic Acid (C22:0)	0.54
Arachidic Acid (C20:0)	0.33
Palmitoleic Acid (C16:1)	0.18
Lignoceric Acid (C24:0)	0.18
Myristic Acid (C14:0)	0.17
a-Linolenic Acid (C18:3n3)	0.15
Eicosenoic Acid (C20:1n9c)	0.10
Lauric Acid (C12:0)	0.08
Heptadecanoic Acid (C17:0)	0.04
Tricosanoic Acid (C23:0)	0.02
Heneicosanoic Acid (C21:0)	0.02

* Cx:y indicates the number of carbon atoms (x) and double bonds (y); n indicates the omega position, and c indicates cis configuration.

**Table 2:** Moisture content and water aging of the films.

Sample	Moisture Content (%)	Water Aging (%)
JW-0	59.10 ± 4.54	88.60 ± 0.40
JW-1	57.14 ± 3.12	83.10 ± 1.03
JW-2	46.90 ± 5.72	80.39 ± 0.68

Results are shown as the mean ± standard error of the mean.

**Table 3:** Thickness and mechanical properties of JW-0, JW-1 and JW-2 films.

Samples	Thickness (mm)	TS (MPa)	YM (MPa)	EAB (%)
JW-0	0.55 ± 0.00 ^a^	8.13 ± 0.03 ^a^	24.19 ± 1.85 ^a^	188.17 ± 5.44 ^a^
JW-1	0.57 ± 0.03 ^a^	6.30 ± 0.15 ^b^	10.14 ± 1.12 ^b^	230.73 ± 2.48 ^b^
JW-2	0.57 ± 0.05 ^a^	5.33 ± 0.09 ^c^	9.62 ± 0.84 ^b^	208.83 ± 3.19 ^c^

^a,b,c^ Different letters in the same column represent significant differences (*p* < 0.05). Results are shown as the mean ± standard error of the mean. TS: tensile strength; YM: Young’s modulus; EAB: Elongation at break.

## Data Availability

The original contributions presented in this study are included in the article. Further inquiries can be directed to the corresponding author.
